# Morphology, ecology, and molecular biology of a new species of giant larvacean in the eastern North Pacific: *Bathochordaeus mcnutti* sp. nov.

**DOI:** 10.1007/s00227-016-3046-0

**Published:** 2016-12-15

**Authors:** R. E. Sherlock, K. R. Walz, K. L. Schlining, B. H. Robison

**Affiliations:** Monterey Bay Aquarium Research Institute, Moss Landing, CA 95039 USA

## Abstract

*Bathochordaeus mcnutti* sp. nov. is described from the mesopelagic northeast Pacific Ocean (Monterey Bay, California, USA). Larvaceans in the genus *Bathochordaeus* are large, often abundant zooplankters found throughout much of the world ocean, but until recently it was unclear whether more than a single species of *Bathochordaeus* existed. Using remotely operated vehicles, we have made hundreds of in situ observations, compiled two decades of time-series data, and carefully collected enough specimens to determine that three species of *Bathochordaeus* occur in Monterey Bay: *B. charon* (Chun), *B. stygius* (Garstang), and *B. mcnutti* sp. nov. *Bathochordaeus mcnutti* is readily distinguished from its two congeners by the distinct blue outline visible around the periphery of its tail, and by other aspects of its morphology, ecology, and genetics. The abundance of larvaceans means they are ecologically important as particle processors. Species within the genus, *Bathochordaeus*, comprise the largest of described larvaceans.

**Electronic supplementary material:**

The online version of this article (doi:10.1007/s00227-016-3046-0) contains supplementary material, which is available to authorized users.

## Introduction

The class Appendicularia, or Larvacea, is composed of three extant families: the Oikopleuridae, the Fritillaridae, and the Kowalevskiidae. Exclusively marine organisms, larvaceans are holoplanktonic pelagic tunicates. As a group, they are important members of the planktonic community, and are capable of having a grazing impact that exceeds that of copepods (Alldredge 1981; Deibel 1988; Fenaux et al. 1998; Gorsky and Fenaux 1998; Lombard et al. 2011). A few species have been well studied, particularly those that are easily kept alive in the laboratory, like *Oikopleura dioica*.

The majority of larvacean species have not been subject to investigation. Larvaceans occur throughout the world ocean, yet their published diversity remains remarkably, and likely inaccurately, low (Hopcroft 2005). Only about 70 species are currently recognized, with 43 of those found in the Pacific Ocean (Fenaux et al. 1998; Castellanos et al. 2009; Deibel and Lowen 2012). In plankton tows, the small species can be very abundant, often rivaling copepods in number, but their fragility makes larvaceans notoriously difficult to collect and study (Bückmann and Kapp 1975; Fenaux 1993). Nowhere is this more clearly the case than in the open ocean and deep sea where the expense and difficulty of sampling are greatly increased (Hopcroft and Robison 1999; Hopcroft 2005).

Consequently, it is likely that numerous groups—larvaceans among them—in these extensive habitats have many species yet to be discovered and formally described (Appeltans et al. 2012). The relative dearth of deep-sea specimens may mean that some described species are in need of more careful examination, particularly those that are cryptic (Pimm 2012). In Monterey Bay, larvaceans of various species are found throughout the water column and the numbers of individuals, if not species (predominantly undescribed) increases as we go through the benthic boundary layer as deep as 3500 m (Robison et al. 2010).

As particle processors, larvaceans affect both the pelagic environment in which they live as well as the seafloor beneath them. The active houses of giant larvaceans function as oases for zooplankton in the mesopelagic habitat (Steinberg et al. 1994) while their abandoned houses, or “sinkers,” are estimated to contribute up to one-third of the vertical carbon flux to the deep seafloor in Monterey Bay (Robison et al. 2005). Because they can transform small, suspended particles into pellets or aggregates that sink quickly, organic carbon processed by larvaceans can shortcut the microbial loop (Deibel 1988; Fenaux et al. 1998). Lombard et al. (2011) showed that the size spectrum of prey for the oikopleurid *Oikopleura dioica* ranges from 0.4 to 20% of their body size—a broader range than most planktivorous organisms—as *O. dioica* feeds on microphagous as well as macrophagous organisms. Resolving the identities of these animals is thus of ecological as well as taxonomic significance.

Giant larvaceans are oikopleurids in the subfamily Bathochordaeinae. Chun (1900) described the first giant larvacean from animals he collected during the *Valdivia* expedition that was made over 100 years ago. He named them *Bathochordaeus charon* after the mythical ferryman who carries the souls of the dead across the River Styx. During the *Valdivia* expedition, two specimens were collected from the South Atlantic and two more from the Indian Ocean. This is noteworthy because of the relative rarity with which these animals are collected using modern trawl equipment (Hopcroft 2005). It was not until three decades after their original description that Garstang (1936) collected two more giant larvaceans in good condition. His specimens differed from those of Chun, and since he had no access to the original specimens, Garstang (1937) rather reluctantly described a new species: *Bathochordaeus stygius*.

Over the subsequent years, giant larvaceans were infrequently collected. When specimens were found, most seemed to fit Garstang’s (1937) description of *B. stygius*. The two species were alternately distinguished then synonymized, with the result that confusion remains in the modern literature over the actual number of extant giant larvacean species (Fenaux 1966, 1993; Flood 2005; Hopcroft 2005; Sherlock et al. 2016).

The advent of tools like remotely operated vehicles (ROVs) and specialized sampling equipment allows us to both observe and carefully collect pelagic specimens, providing the opportunity to better study the diversity of fragile organisms like larvaceans. We have made thousands of in situ observations and numerous collections of these delicate animals. Nearly two decades of data from MBARI’s Mesopelagic Time Series have provided insight into their ecology and distribution. From these data, we have learned that *Bathochordaeus* exhibits a seasonal peak in abundance in Monterey Bay and has a reproductive strategy that differs markedly from that of other oikopleurids.

Within our video recordings, the presence of a vivid blue line surrounding the tails of some *Bathochordaeus* suggested that there were at least two different forms of giant larvaceans in Monterey Bay. However, carefully collected specimens have allowed us to make detailed laboratory observations and to use modern molecular techniques in order to determine that, in fact, three giant larvacean species occur in Monterey Bay. The most common species is *Bathochordaeus stygius* Garstang (1937). A second much less common species fits well with Chun’s (1900) original description of *B. charon* (Sherlock et al. 2016). The third is a new species of giant larvacean in the genus *Bathochordaeus* that we describe here based on morphological, ecological, and molecular evidence: *Bathochordaeus mcnutti* sp. nov.

## Materials and methods

The Monterey Bay Aquarium Research Institute (MBARI) has relied upon three ROVs, *Ventana*, *Tiburon*, and *Doc Ricketts*, to make sample collections and video observations of fragile, pelagic organisms since 1988. All three ROVs have been equipped with either standard or high-definition video cameras (Robison 1993; Robison et al. 2010). Between them, these vehicles have amassed close to 22,000 h of video footage, annotated by biologists and organized in a database called VARS, the Video Annotation and Reference System (Connor 2005; Schlining and Stout 2006).

A mesopelagic time series for Monterey Bay that spans approximately two decades was used to compare ecological data for both types of *Bathochordaeus*. During ROV dives, data for depth (m), temperature (°C), salinity (psu), and oxygen concentration (*ml/l*) were recorded continuously with sensors installed on the ROVs. These data are merged with each annotated record of *Bathochordaeus* spp. in VARS and provide in situ environmental data for all observations.

Giant larvaceans are often visible from several meters away because of the outer filter or “house” that surrounds them, which is often greater than a meter in longest dimension (Barham 1979; Hamner and Robison 1992). The blue outline on the tail of *Bathochordaeus mcnutti* is difficult to see if the animal is more than a meter away from the ROV’s camera, and specimens close to the camera may be difficult to see clearly if the ROV is moving fast. Therefore, only video footage that was clear and close-up was utilized for enumeration and to determine the depth distribution of *B. mcnutti* and *B. stygius*.

Physical descriptions are based on in situ observations made from 1991 to 2014 and on laboratory examinations of dozens of specimens collected over that time period. Preservation for morphological examination was typically done with 5% formalin; however, cacodylate-buffered 2% glutaraldehyde was occasionally used as well. All measurements reported here were made on formalin-preserved animals. The type locality for holotype and paratype specimens of *B. mcnutti* sp. nov., and two congeners are given (Table 1).

**Table 1:** Collection information for type specimens of *Bathochordaeus mcnutti* sp. nov., *B. stygius* (Garstang 1937) and *B. charon* (Chun, 1903)

Specimen	Date	Dive#	ROV	Depth (m)	Lat.	Long.	USNM#
*B. mcnutti*	8/20/2013	3730	Ventana	135	36.7587	−122.1042	1422057
*B. mcnutti*	8/20/2013	3730	Ventana	99	36.7595	−122.1033	1422056
*B. mcnutti*	8/20/2013	3730	Ventana	93	36.7603	−122.1028	1422058
*B. mcnutti*	8/27/2009	3410	Ventana	91	36.7495	−122.1050	1422059
*B. charon*	11/11/2013	548	Doc Ricketts	598	36.5409	−122.5205	1251906
*B. charon*	3/28/2013	457	Doc Ricketts	281	36.6887	−122.0438	1251907
*B. stygius*	8/20/2013	3730	Ventana	285	36.7507	−122.1033	1251908
*B. stygius*	8/20/2013	3730	Ventana	270	36.7544	−122.1055	1251909
*B. stygius*	11/18/2013	545	Doc Ricketts	215	36.7480	−122.1022	1251910

The catalog numbers (USNM #) for the Smithsonian Institution, National Museum of Natural History are given

For molecular work, animals were placed in 95% ethanol or liquid nitrogen for initial preservation and stored at −80 °C until extraction. Samples of DNA were extracted from tissue using the DNeasy^®^ Kit (Qiagen, Valencia, CA, USA) according to the manufacturer’s instructions. Traditional DNA barcoding primers such as those for cytochrome c oxidase 1 (Folmer et al. 1994), 12S, cytochrome b, and H3 did not amplify genes for *Bathochordaeus* spp., and therefore we selected two tunicate primers used by Hirose et al. (2009), that were constructed specifically for tunicates, salps, doliolids, and larvaceans:

5′-CATTTWTTTTGATTWTTTRGWCATCCNGA-3′ (UroCox1-244F).

5′-GCWCYTATWSWWAAWACATAATGAA ARTG-3′ (UroCox1-387R).

These primers amplified a 400-base-pair section of the mitochondrial cytochrome *c* oxidase subunit I (COI) gene with the following PCR parameters: 35 cycles of 94 °C for 1 min, 40 °C for 1 min, 72 °C for 1 min. Amplification of an 1800-base-pair fragment of small subunit ribosomal DNA (18 S) was done using the modified universal primers *mitchA* and *mitchB* from Medlin et al. (1988) with the following PCR parameters: 4 cycles of 94 °C for 1 min, 54 °C for 1 min, a step of 0.1 °C/second from 54–72, 72 °C for 2 min, followed by 29 cycles of 94 °C for 1 min, 58 °C for 1 min, 72 °C for 1:30 min. All products were bidirectionally sequenced using BigDye^®^ Terminator v3.1 Cycle Sequencing Kit on an ABI 3100 or ABI 3500xl Genetic Analyzer (Applied Biosystems, Foster City, CA, USA). Sequences were edited and aligned using Geneious version 6.0.5 created by Biomatters (Auckland, New Zealand, http://www.geneious.com/), and submitted to GenBank (Accession Numbers: KX599256-KX599281). Haplotype networks were created in TCS v1.21. Distance matrix was generated in Mega v6.

## Results

### Systematics

- OIKOPLEURIDAE Lohmann, 1915
- *Subfamily Bathochordaeinae* Lohmann, 1915
- *Bathochordaeus* Chun, 1900: 519 (Redescribed in Fenaux and Youngbluth 1990: 759)
- TYPE SPECIES: *Bathochordaeus charon* Chun 1900: 519
- TYPE SPECIES: *Bathochordaeus stygius* Garstang 1937: 283
- *Bathochordaeus mcnutti*, new species

### Type material

The holotype, collected from a depth of 99 m (where the seafloor is 1058 m) in Monterey Bay, California (36.759570°N, −122.103366°W) is deposited at the Smithsonian Institution, National Museum of Natural History, USA (catalog number USNM 1422056).

### Diagnosis

Adult giant larvaceans in the genus *Bathochordaeus* are larger than most other adult larvaceans by close to an order of magnitude. In our samples, adult specimens (based on the presence of eggs and testes) range in size from approximately 3–10 cm in total length. From a distance in situ, size can be a distinguishing characteristic for the genus. Observed under a dissecting microscope, even a small *Bathochordaeus* specimen is readily separated from all other oikopleuridae by its asymmetrical mid-dorsal field cells, i.e., the large cells in the oikoblastic layer on either side of the animal’s midline are not mirror images of each other (Flood and Deibel 1998).

**Fig. 1:**
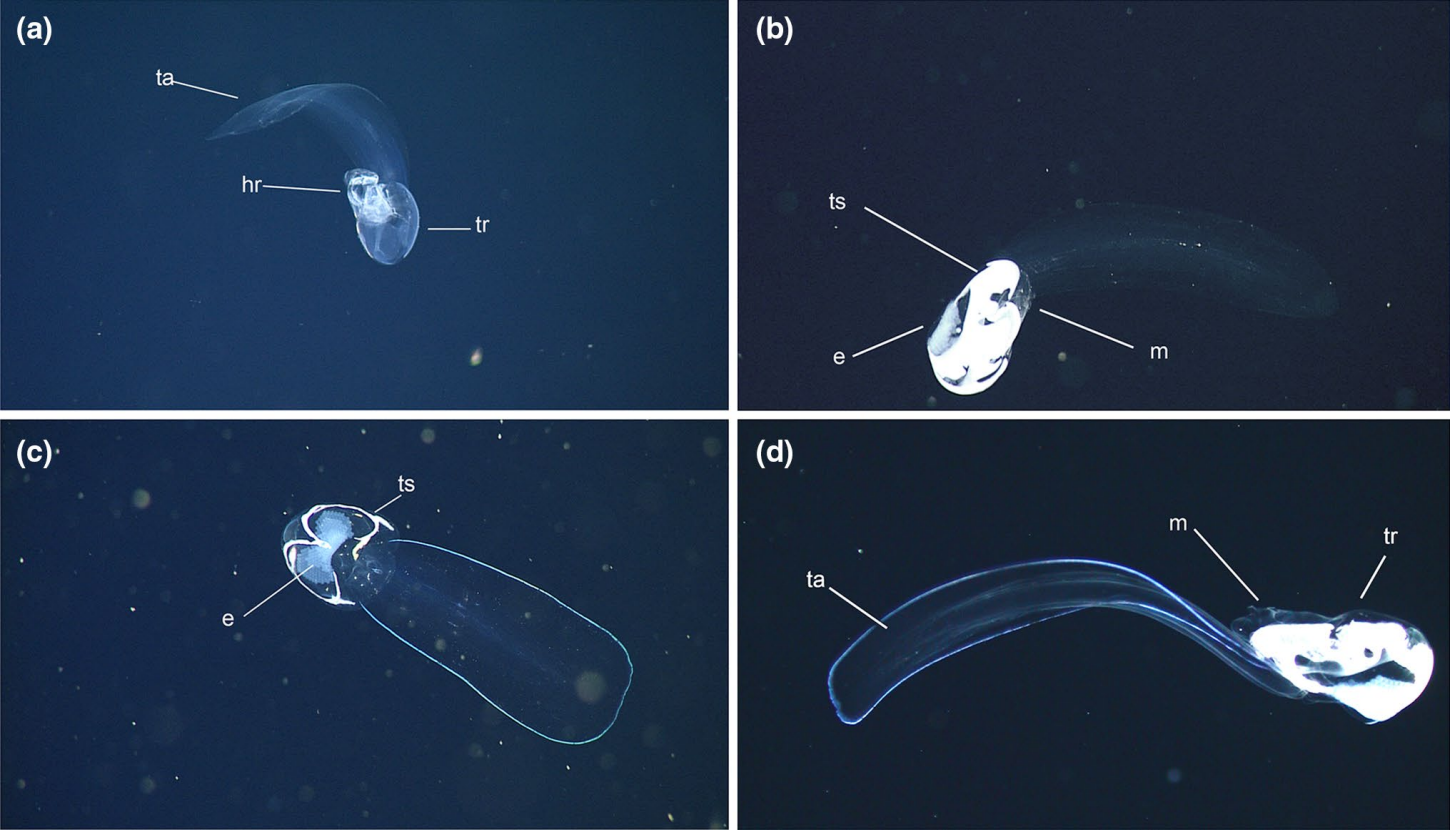
In situ comparison of *B. stygius* (**a**, **b**) to *B. mcnutti* sp. nov. (**c**, **d**). The tail (ta) and trunk (tr) of all specimens are clearly visible. One animal is beginning to inflate its “house” (house-rudiment), (h) but the rest are fecund adults with well-developed testes (ts), and eggs (e), that no longer occupy a house

**Fig. 2:**
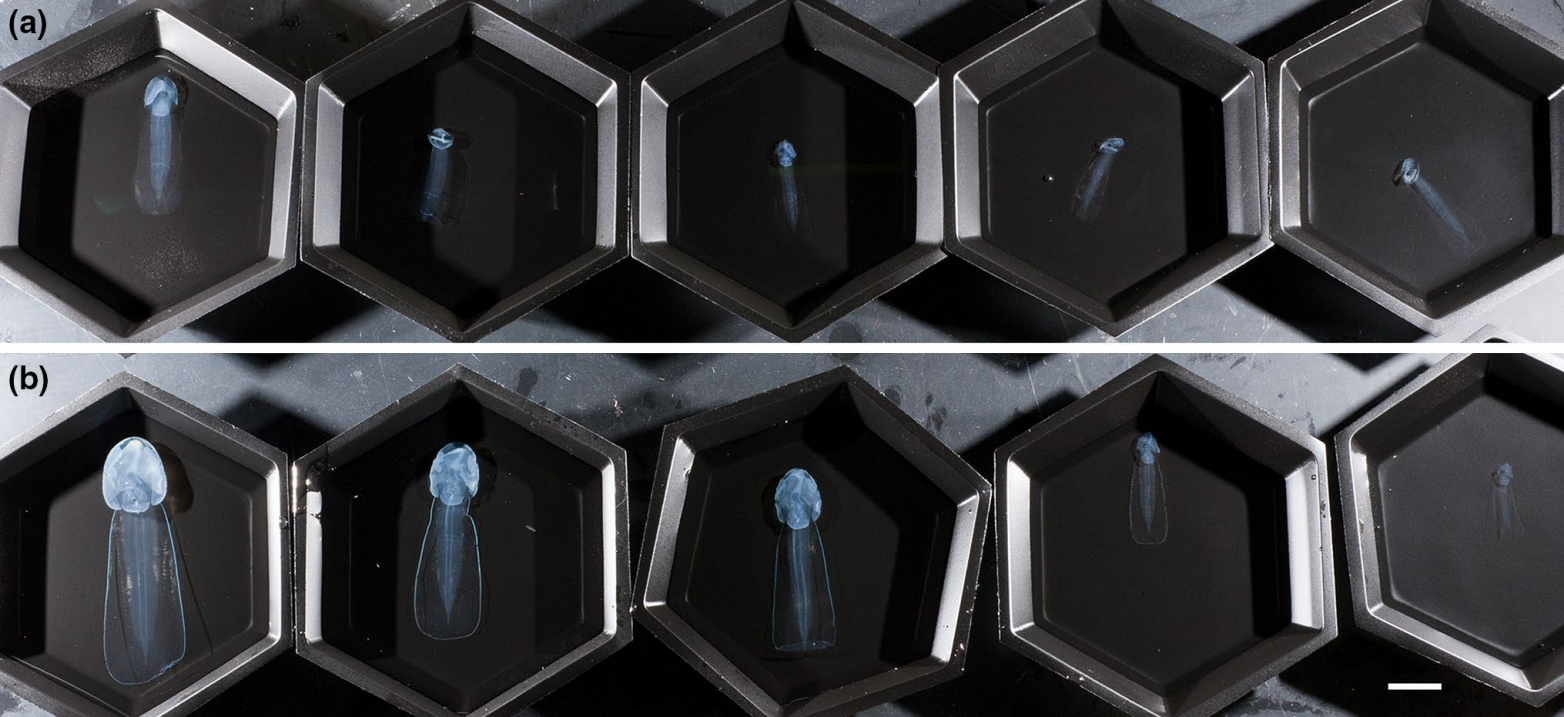
Comparison of *B. stygius* (*top row*, **a**) and *B. mcnutti* sp. nov. (*bottom row*, **b**) preserved in 5% formalin. All 10 specimens were collected by ROV (remotely operated vehicle) on August 30, 2013. The outline around the tail of *B. mcnutti* has lost its blue coloration but remains readily apparent over the large size-range of specimens (*scale* 2 cm)

The shape and size of the houses of *B. mcnutti* and *B. stygius* observed in situ are very similar to each other but are quite different from *B. charon* (Sherlock et al. 2016). Unless gravid, the trunk (body) and tail of *Bathochordaeus* species in Monterey Bay are largely transparent. The most conspicuous pigmentation in *B. stygius*, if present, occurs in the gut and varies with the food consumed. In contrast, specimens of *B. mcnutti* bear a striking, iridescent blue outline around the periphery of their tails (Fig. 1). This is visible in the lights of a ROV but also under a microscope and in daylight. The outline remains visible even after preservation in formaldehyde, although the blue color turns an opaque white (Fig. 2). When examined closely, key differences in the morphology of their pharynx, spiracles, and esophagus combine to further distinguish *B. mcnutti* from its congeners.

### Etymology

The specific name *mcnutti* serves as an honorific to Marcia McNutt, Ph.D. MBARI’s President/CEO at the time this species was discovered.

*Orientation to the animal:* The mouth of *Bathochordaeus* opens dorsally, near the anterior end of the animal and the anus voids ventrally just right of the midline and behind the spiracles. The trunk of the animal embodies the mouth, spiracles, both lobes of the stomach, the digestive tract, gonads, brain, and heart. In comparison, the tail appears almost without structure and attaches to the trunk ventrally.

**Fig. 3:**
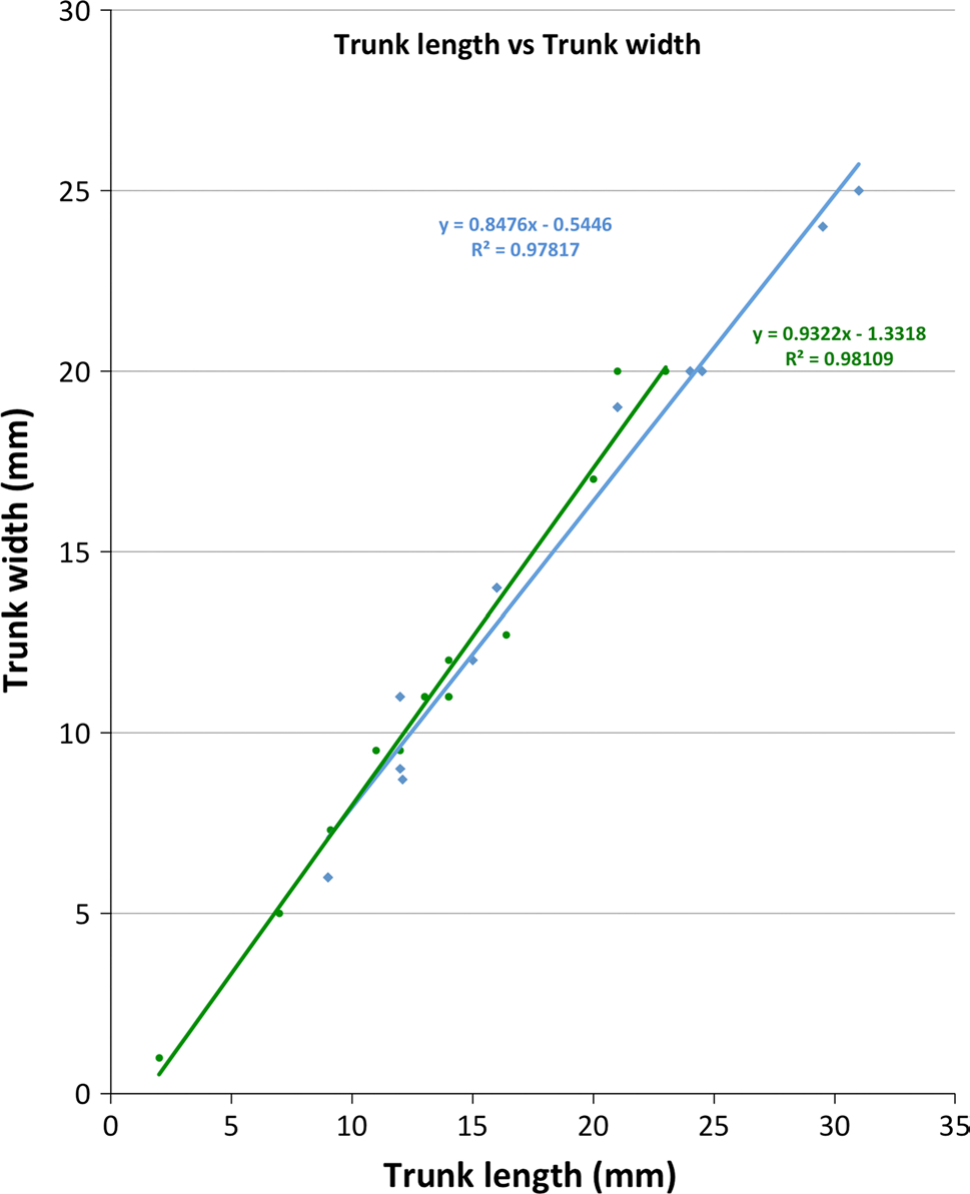
Trunk length versus width. Measurements were made on preserved (5% formalin) specimens of various sizes (*n* = 10 *B. mcnutti*, 10 *B. stygius*). For both species, the trunk is slightly longer than wide

*Trunk*: The trunk of *Bathochordaeus mcnutti* is slightly longer than wide (1.2:1.0) as is that of *B. stygius* (Fig. 3), and the trunks of both species appear oval when viewed from above or below (Fig. 2). The height of the trunk tapers and at its thickest, most posterior point, it is slightly less thick than wide. The anterior end of the trunk is narrower by approximately half. These features combine to give the trunk a blunt, slightly wedge-shaped profile that is similar for both species (Fig. 1).

*Tail*: Blue outline encircles the periphery of the entire tail of *B. mcnutti* (Figs. 1, 2). The broad tail widens as it progresses from the trunk to its distal end, at which point it is blunt with rounded corners.

**Fig. 4:**
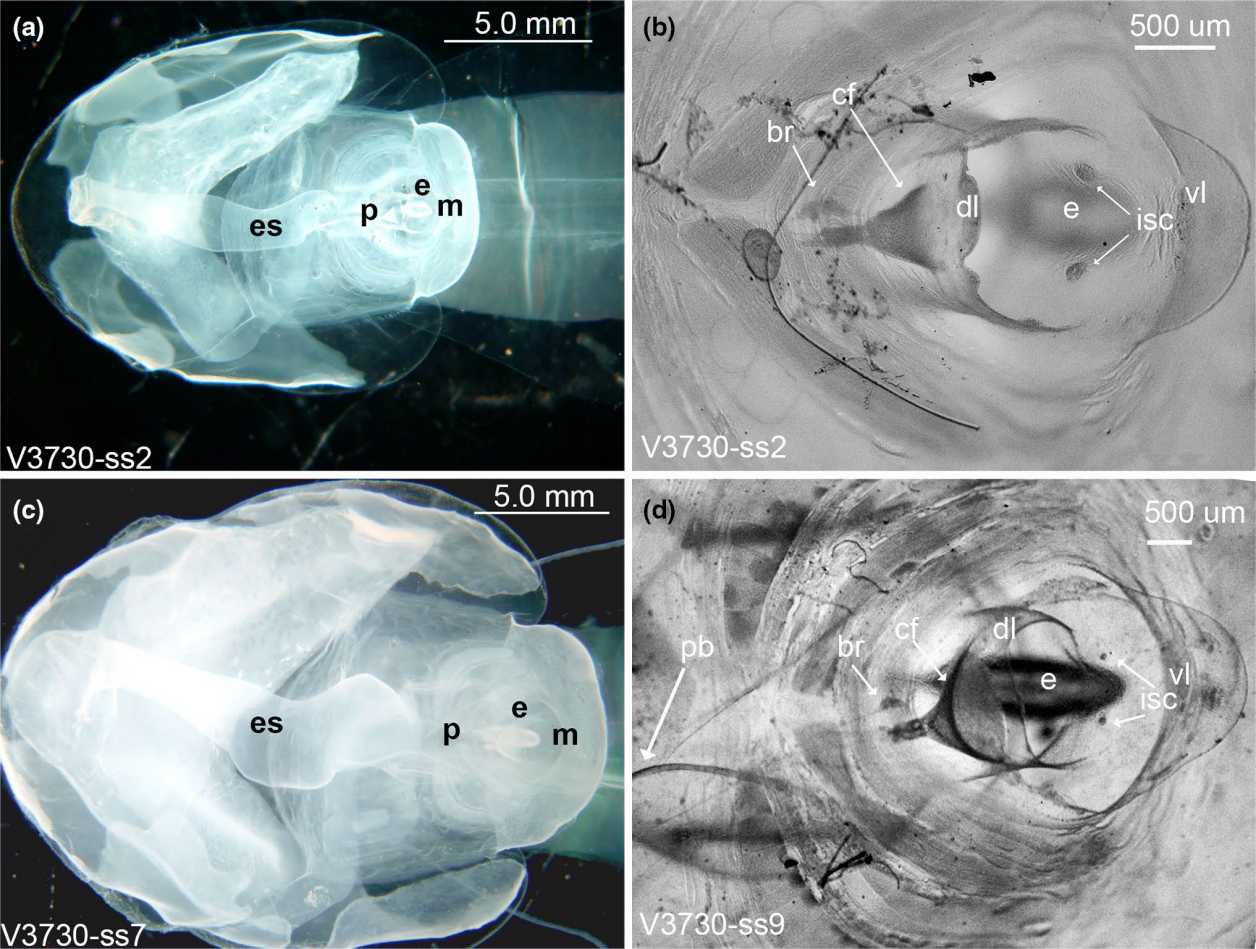
Dorsal views of *B. stygius* (*top row*, **a**, **b**) and *B. mcnutti* sp. nov. (*bottom row*, **c**, **d**). In *B. mcnutti*, the pharynx (p) is broad and the esophagus (es) appears distinctly kinked (**c**). Both structures are more slender in *B. stygius*, and the esophagus is almost tubular (**a**). To accommodate the bulkier pharynx, the endostyle (e) of *B. mcnutti* is pushed down, closer to the floor of the trunk. Its more horizontal aspect makes the endostyle of *B. mcnutti* appear elongate in comparison with *B. stygius* when viewed from above or below. In both species, the ventral lip (vl) is pronounced (**b**, **d**). The dorsal lip (dl) of *B. mcnutti* is generally smooth and uniform (**d**), while that of *B. stygius* usually looks more scalloped, due in part to the presence of densely packed cells at its apex (**b**). These same cells are less conspicuous in *B. mcnutti* and may be absent, entirely (**d**). The ciliated funnel (cf) of *B. mcnutti* (**d**) is typically more pronounced than that of *B. stygius* (**b**) because it is usually larger relative to the mouth (m). The inner sensory cells (isc) lie just below the ventral lip and in *B. mcnutti* (**d**) are small relative to *B. stygius* (**b**) of similar size and may be absent. Specimen identification numbers begin with “V” for the ROV *Ventana* and are followed by the dive number. In plate (**b**) a fiber is attached to the trunk and is not part of the specimen

*Mouth and lips*: The dorsal lip of *B. mcnutti* is semi-circular, smooth or very slightly scalloped (Fig. 4). The dorsal lip of *B. stygius* is usually distinguished from *B. mcnutti* because the former typically has a prominent band of densely packed cells, just above the ciliary funnel (cf) sometimes giving the dorsal lip a slightly scalloped look that Garstang referred to as “corrugated” (Fig. 4). More rarely, we have observed similar cells along the dorsal lip of *B. mcnutti*, but when present, they are not so densely packed as in *B. stygius*. In *Oikopleura dioca*, similar cells aid in particle rejection by causing ciliary reversal in the spiracles (Galt and Mackie 1971; Fenaux 1986; Bassham and Postlethwait 2005), but in *B. stygius* their function is not known. The conspicuous ciliated funnel itself is a prominent pharyngeal feature just below the dorsal lip, and it lies in approximately the same position for *B. mcnutti* as *B. stygius*, just right of the midline and brain. However, the ciliated funnel of blue-tailed larvaceans usually appears larger relative to their mouth than does that of *B. stygius* because the ciliated funnel of *B. mcnutti* is approximately two-thirds the diameter of the mouth while that of *B. stygius* is generally only half the diameter of the mouth or less (Fig. 4). Both of these species are markedly different in the orientation and aspect of the ciliated funnel than their congener *B. charon* (Sherlock et al. 2016). The ventral lip is pronounced in the genus, *Bathochordaeus*, but the inner sensory cells just below it are diminished in *B. mcnutti* compared to *B. stygius* and may even appear to be absent in smaller *B. mcnutti* (Fig. 4). The function of the inner sensory cells is unknown, but they seem likely to also play a role in particle rejection.

**Fig. 5:**
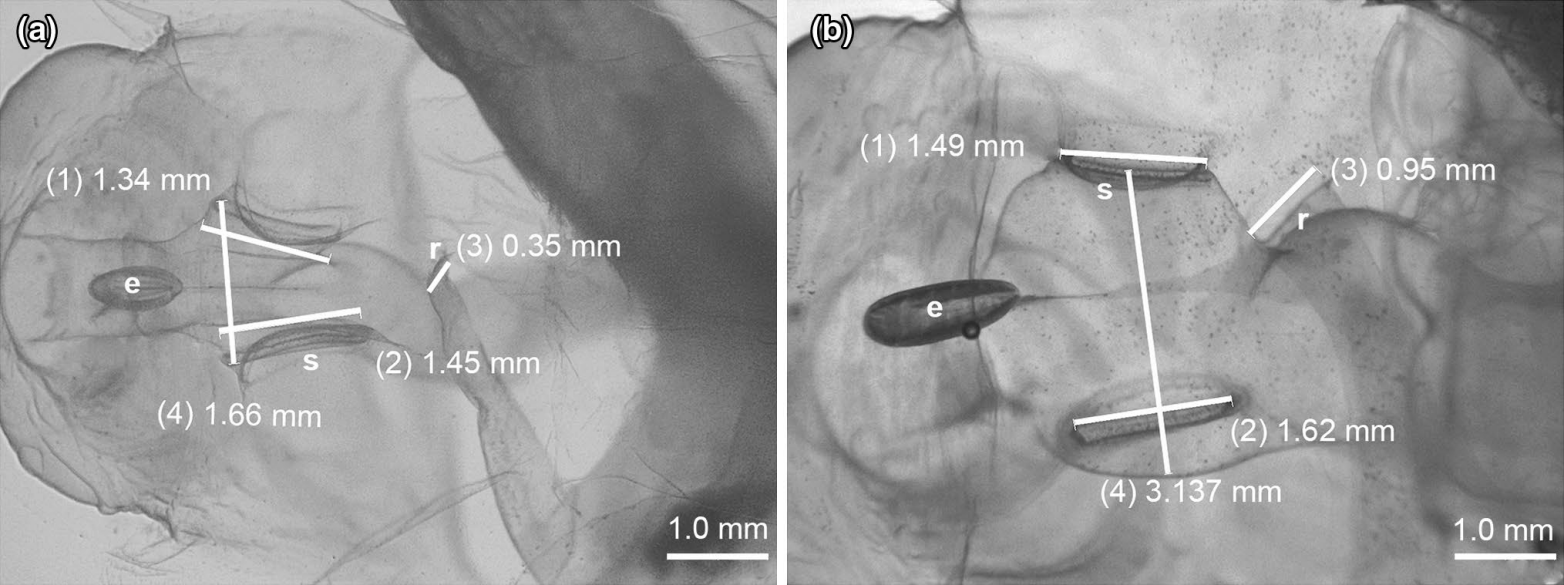
Ventral view of the trunk. The inner, pharyngeal openings to the spiracles (s) of *B. stygius* (**a**) and of *B. mcnutti* sp. nov. (**b**) are approximately equal in diameter to their outer openings. The spiracles of *B. mcnutti* (**b**: 1, 2) are about half the diameter of the pharynx at their midpoint (4). The slightly cupped spiracles of *B. stygius* (**a**: 1, 2) are more dorsoventrally flattened and in widest diameter approximate the diameter of the pharynx (4). Observed ventrally, the endostyle (e) of *B. mcnutti* appears more elongate than that of *B. stygius*. The rectum (r) voids slightly left of the mid-line in each species

*Pharyngeal region* (*including endostyle and esophagus*): The pharynx of *B. mcnutti* is larger and more robust than that of *B. stygius* (Fig. 4) and is encircled by peripharyngeal bands that when viewed dorsally, describe a distinct hourglass shape before taking a slightly convoluted path to the esophagus. On the other hand, the pharynx of *B. stygius* is narrow in comparison and gracefully follows a straighter path to its more tubular esophagus. Where the pharynx meets the esophagus of *B. mcnutti*, it appears kinked, from above angling briefly right at its junction with the pharynx, then veering to the left as the esophagus continues toward the stomach (Figs. 4, 5). In both species, there is a junction where the peripharyngeal bands meet dorsally, prior to the twisting of the pharynx, but in *B. mcnutti*, their sinuous outline contributes to the Fig. 8 like shape of the pharynx (online supporting video). Perhaps in order to accommodate its bulkier pharynx, the endostyle of *B. mcnutti* appears to have rotated from the more vertical (dorsal–ventral) orientation of *B. stygius* to a more horizontal (posterior–anterior) orientation, such that when viewed directly from above or below, the endostyle appears quite long relative to that of *B. stygius* (Figs. 4, 5).

*Spiracles*: The diameter of the spiracles is approximately equivalent at inner and outer openings for *B. mcnutti* and *B. stygius* (Fig. 5), i.e., neither species has funnel-shaped spiracles like *B. charon* (Sherlock et al. 2016). However, blue-tailed larvaceans have conspicuously smaller spiracles relative to the width of their pharynx than does *B. stygius*, and the spiracles of *B. mcnutti* appear more circular and less dorsoventrally flattened than those of *B. stygius.* In part, this is due to the robust pharynx of *B. mcnutti* versus the more slender, almost tubular pharynx of *B. stygius*. In longest dimension, the spiracle diameter of *B. stygius* exceeds the width of the pharynx at its mid-point. The diameter of the spiracles of *B. mcnutti* is clearly less than the width of the pharynx at their mid-point. When viewed either dorsally or ventrally, the outer spiracular opening of *B. stygius* is slightly cupped and appears flared. In contrast, the outer openings of the spiracles of *B. mcnutti* appear more flat than cupped (Fig. 5).

**Fig. 6:**
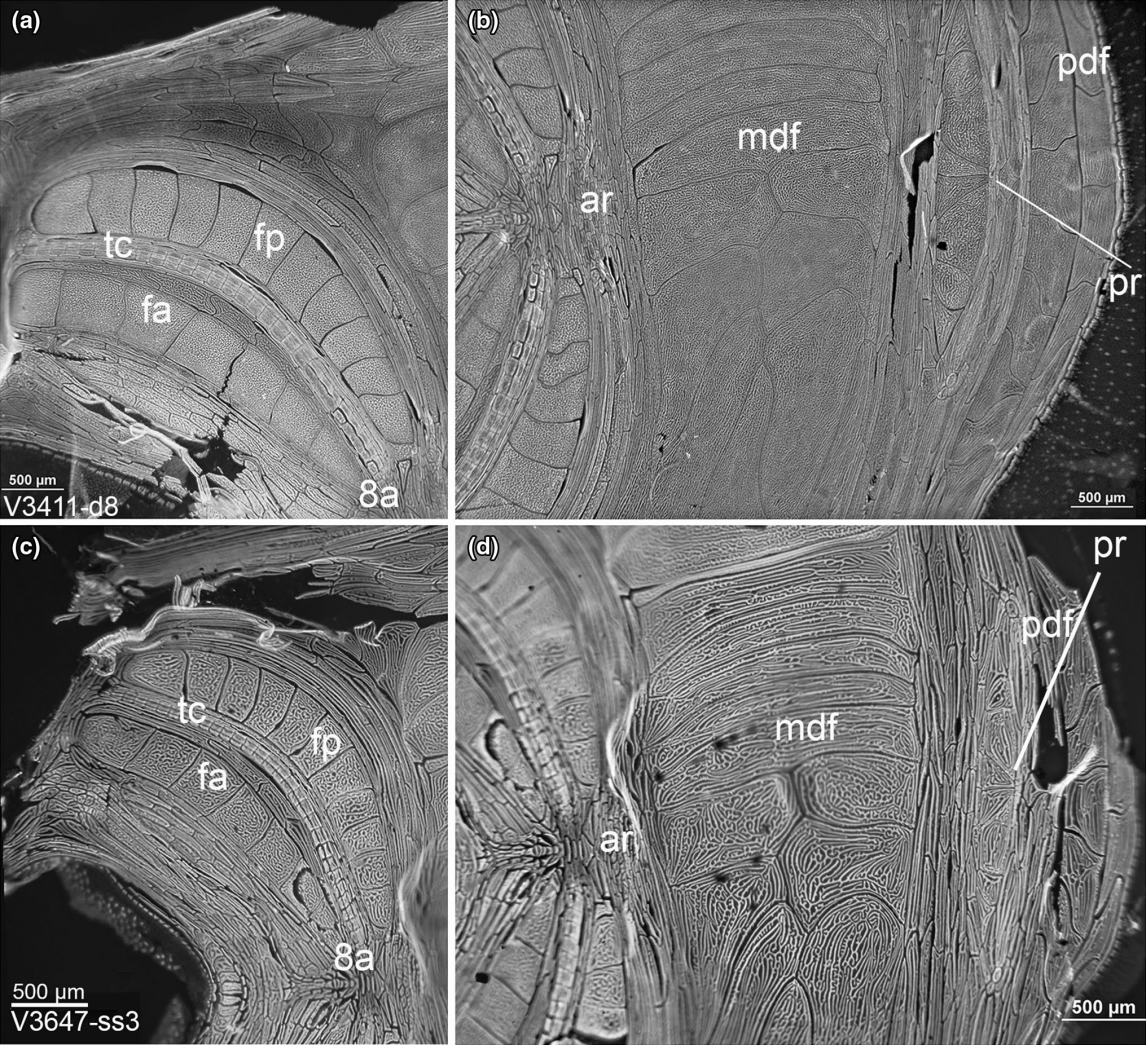
DAPI-stained oikoblastic regions of *Bathochordaeus stygius* (**a**, **b**) and *B. mcnutti* sp. nov. (**c**, **d**) continue somatic growth after cell division ends; thus, the cells of which it is comprised are large and many times polyploid. Two conspicuous, crescent-shaped bands on either side of the mouth form Fol’s oikoplast. There are eight anterior Fol’s cells (fa) and 12 posterior cells (fp) that, with the trap cells (tc, also called “fibroblasts”), make the inner food-concentrating filter. Posterior to Fol’s oikoplast is the mid-dorsal field (mdf) that is composed of even larger cells. Unique to the genus *Bathochordaeus*, these cells are not bilaterally symmetrical. Since the oikoblastic cells are responsible for making the house with which these animals live and feed, it is expected that differences in the shapes of these cells will give rise to differences in the house structure. *Scale* 500 µm all plates

*Oikoblastic region*: The number of oikoblastic cells of all oikopleurids is thought to be constrained after the tail shift made during development (Lohmann 1898; Fenaux 1998b). However, the oikoblastic cells continue to grow as the animals age, and DNA replicates. This makes the oikoblastic cells of oikopleurids many times polyploid (Flood 2005). With *Bathochordaeus*, this endopolyploidy results in particularly large oikoblastic cells (Fig. 6). However, despite their size the oikoblastic cells are often difficult to see because they are colorless and can be easily damaged, and also because healthy *Bathochordaeus* spp. will begin to make a house very soon after capture, and the oikoblastic region can be obscured by the house-rudiment. Staining with DAPI or Hoechst 33258, both of which have an affinity for DNA, makes the polyploid cells of the oikoblastic region easily visible with a dissecting microscope, when illuminated by ultraviolet light.

The most conspicuous cells of the oikoblastic region are the two pairs of dorsal bands that border either side of the mouth of *Bathochordaeus*, that Garstang called “moustache-shaped,” some cells of which can be close to a millimeter in longest dimension (Fig. 6). With the triple rows of evenly spaced, and much smaller trap cells in between, these paired bands comprise Fol’s oikoplast. In number, the giant cells of Fol’s oikoplast do not differ between *B. mcnutti* and *B. stygius*: eight for the anterior crescent and 12 for the posterior crescent. Chun (1900) described these as “moustache-like,” although he may have confused the anterior crescent with trap cells (or “fibroblasts”), because the anterior crescent of *B. charon* is greatly reduced in size compared to its congeners and is more similar in size to the band of much smaller (but more numerous) fibroblasts (Sherlock et al. 2016). When he described *B. stygius* with their much larger anterior Fol’s cells, Garstang (1937) was perplexed at how both Chun (1900) and Lohmann (1914) failed to see them when describing *B. charon*. In large part, these differences caused Garstang, reluctantly, to describe *B. stygius* in the first place. However, he thought that the posterior crescent was a modification of Martini’s field and that Lohmann was mistaken in calling them Fol’s cells. Instead Garstang (1937) referred to them as “Lohmann’s colloplasts” (Fig. 6). In most other oikopleurids, there are eight large Fol’s cells that are responsible for making the inner feeding filter of the house. The food-concentrating filters of *B. mcnutti* as well as *B. stygius* are made by all four bands of dorsal giant cells, and so it seems logical to refer to them as Fol’s posterior and Fol’s anterior when designating the twelve and eight-cell bands, respectively.

Behind Fol’s oikoplast are the even larger mid-dorsal field cells. Here the genus *Bathochordaeus* differs from all other oikopleurids in that the mid-dorsal field (mdf) is not bilaterally symmetrical. Apparently this difference was not noticed by Chun and was not well described by Lohmann (Garstang 1937). The posterior-dorsal field (pdf) is sandwiched between the mdf and the posterior rosette (also called Lohmann’s cross) that, at least for *Bathochordaeus* spp, looks neither like a cross nor a rosette. In number, the cells of the oikoblastic region appear similar for the two species, if not identical. But between *B. stygius* and *B. mcnutti*, there are consistent, if subtle differences in the whorls and interstices surrounding those cells (Fig. 6). Flood (2005) speculated that the patterns of cells in the oikoblastic region could provide a means of distinguishing between species, and we would agree; however, the importance of such distinctions is unclear. Such detailed examination of cellular arrangement requires time and equipment that may be outside the scope of many researchers (Hopcroft 2005), particularly when there are more conspicuous morphologic features that are also more clearly definitive, e.g., the digestive tract, spiracles and the blue outline of the tail.

*House*: Chun (1900) speculated that such large animals as *Bathochordaeus* must make “pumpkin”-sized houses. Barham (1979) first observed large larvaceans in their houses from a submersible and estimated their size at 30–100 cm. House size varies and is at least somewhat dependent on the size of the animal, but the giant larvaceans of Monterey Bay produce approximately one house per day (Silver et al. 1998; Robison et al. 2005) and that house can exceed a meter in greatest dimension (Hamner and Robison 1992).

**Fig. 7:**
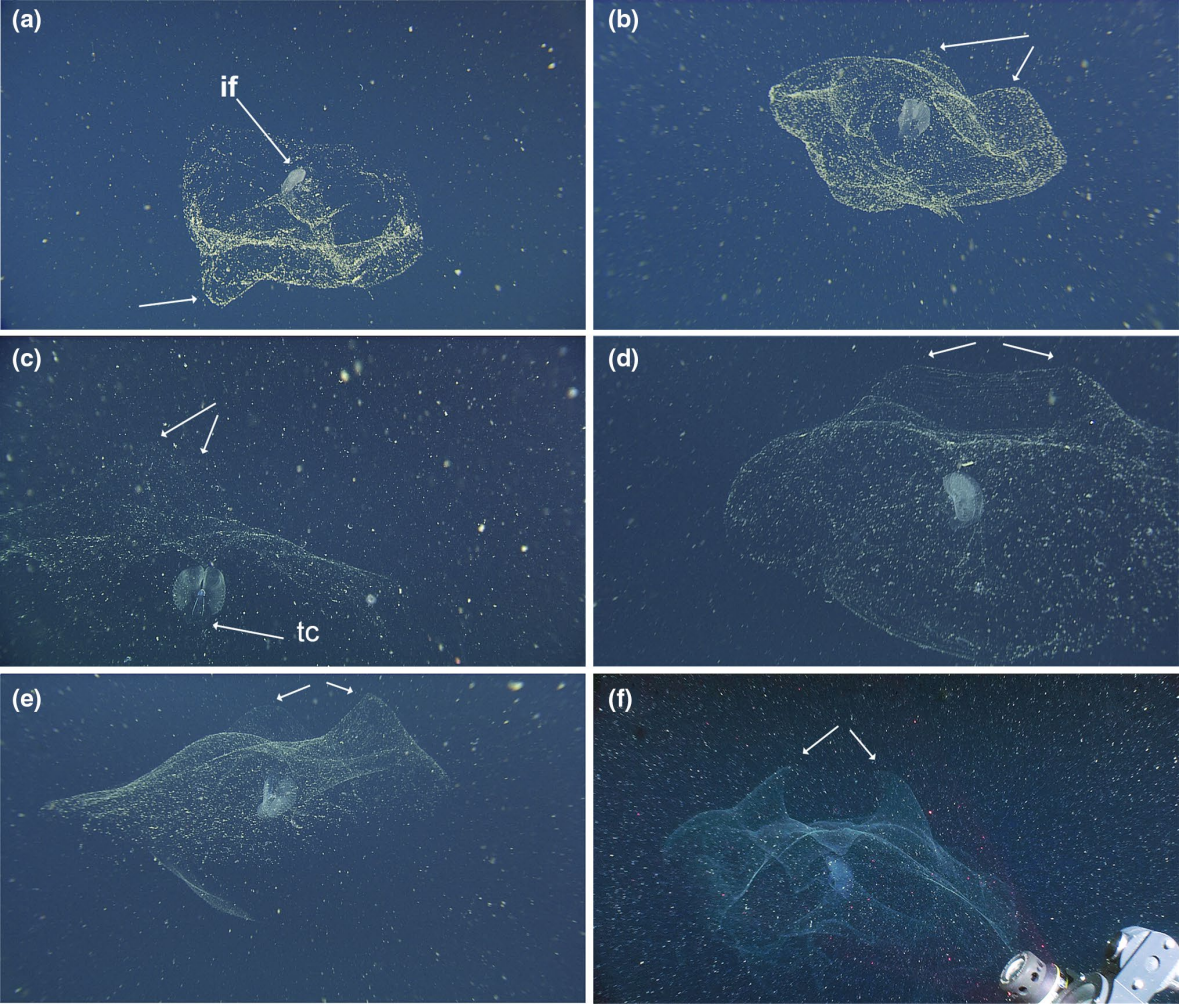
Occupied, undisturbed houses of *Bathochordaeus stygius* (**a**, **b**) and *B. mcnutti* sp. nov. (**c**–**f**). The inner filter (if) is labeled (**a**) and appears in all plates as the relatively opaque, central structure. When recently formed (**c**) the outer house lacks a thick covering of marine snow but it becomes more visible as detritus accumulates, until the point where the outer house may begin to change shape or slough off, sometimes leaving the still-occupied inner filter. Depending on the vantage point, the undistorted houses of both species often show fairly conspicuous protrusions or humps (*arrows*). In (**d**) there is a continuous ridge. Their function is unknown, but these humps are typically more pronounced for *B. mcnutti* than *B. stygius*. In all plates except **a** and **e** the tail chamber (tc) of the animal is oriented in the same direction as the protrusions on the outer houses

The oikoblastic cells of *Bathochordeus* spp. secrete the filters, or “houses” with which these animals feed. The house is acellular, made of mucopolysaccharides and consists of distinct inner and outer structures, with the innermost functioning to concentrate the larvacean’s food, and connected to the animal via a tube made of the same material as the house (Flood and Deibel 1998). Newly inflated houses are so transparent as to be almost invisible. With time, the house becomes easier to see as particles from the surrounding water accumulate and adhere to it, either passively as marine snow or actively as the house filters the water pumped through it (Silver et al. 1998). The inner filter (or filters) is bilobed and bilaterally symmetrical and also becomes clearly visible once filtered particles begin to adhere to it (Fig. 7).

The outer part of the house provides a protective barrier for the inner filter. Steinberg et al. (1994) showed that up to an order of magnitude more metazoans were found on houses than in surrounding waters. Some of these metazoans use the house as substrate. Invertebrates like medusae, ctenophores, and polychaete worms are commonly found trapped in the outer house. In the occupied houses of most other oikopleurids, some metazoans and large particles would be kept out of the inner food-concentrating filters by inlet filters, secreted by Eisen’s oikoplast (Flood and Deibel 1998); however, no Eisen’s cells have been reported in the genus *Bathochordaeus*. Despite this, the outer structure of the house does function as a roof-like barrier between the inner filter and the perpetual rain of marine detritus, as it filters large particles out of the water column and helps prevent metazoans from contacting and damaging the inner filter. As the outer house is coated and covered with particles, it begins to change shape.

The fragility of the outer house, along with the difficulty of observing one in situ makes the outer house a poor character by which to distinguish *B. mcnutti* from *B. stygius*. Nevertheless, one difference seen in the occupied and undisturbed outer house of *B. mcnutti* is a pair of prominent bumps, or a ridge, that if seen in *B. stygius*, is much more subtle (Fig. 7).

In contrast, the inner filter is well defined, more robust and more stereotypical in shape. However, the subtle differences in oikoblastic cells between *B. mcnutti* and *B. stygius* do not appear to generate differences in the inner filters of the houses we have studied, at least no apparent differences that can be discerned from the high-definition ROV video (online supporting video), although both differ markedly from the inner filter of *B. charon* (Sherlock et al. 2016).

*Genital region*: Larvaceans in the genus *Bathochordaeus* are simultaneous hermaphrodites. For the genus, gametogenesis begins near the midline of the ventral floor, close to the posterior end of the trunk. The eggs ultimately develop ventro-laterally while the testes spread posteriorly in wing-like tendrils that end up almost surrounding the eggs, as they expand dorso-laterally to envelop much of the trunk. During gametogenesis, the testes change from being virtually transparent (Fig. 1a) to an opaque white when mature (Fig. 1b–d). Among oikopleurids, gonad shape varies between genera and even between species (Fenaux 1998a). For *Bathochordaeus* spp., the size and wing-like shape of testes vary individually and perhaps between species (Fig. 1). The largest eggs we have measured have been 875 *u*m in diameter, and we have counted as many as 640 eggs from one individual, although the number of eggs is likely relative to the size of the animal and/or the concentration of food present (Paffenhöfer 1973; Fenaux 1998a).

### Spawning and reproduction

All oikopleurids except *Oikopleura dioca* are hermaphrodites (Fenaux 1998a). At least some abandon a final house, swim to the surface to spawn, and then die as gametes escape through their ruptured trunks (Alldredge 1982). In contrast to the smaller species, *Bathochordaeus mcnutti* and *B. stygius* abandon a final house then descend to darker depths, perhaps because their opaque, gamete-laden trunk is less apt to be seen by visual predators. Ultimately, it seems probable that gravid individuals make a final ascent in order to spawn in the shallower waters where they feed, but we have no observations of *Bathochordaeus* spp. spawning in situ. While it is possible that giant larvaceans descend and then spawn at depth, it seems unlikely that they would do so. Captured specimens have spawned in our samplers as the ROV ascended. The majority of eggs from those animals, kept in the water they were collected with, are near-neutral with a small percentage being either very slightly buoyant or sinking slowly. If giant larvaceans spawned at depth, it is difficult to fathom how the young would ascend the hundreds of meters necessary to reach the depth range of 100–300 m where they are most abundant. It is difficult to envision an adaptive benefit to spawning at depth. Furthermore, while we have sampled small individuals in the shallow mesopelagic and epipelagic from 50 to 150 m, below 450 m we have only encountered gravid or spent adults. We have also observed and collected large, gravid individuals in the shallow mesopelagic. Since these animals were swimming free of their houses, they were not feeding and it follows that they were ascending to spawn in the more productive surface waters.

**Fig. 8:**
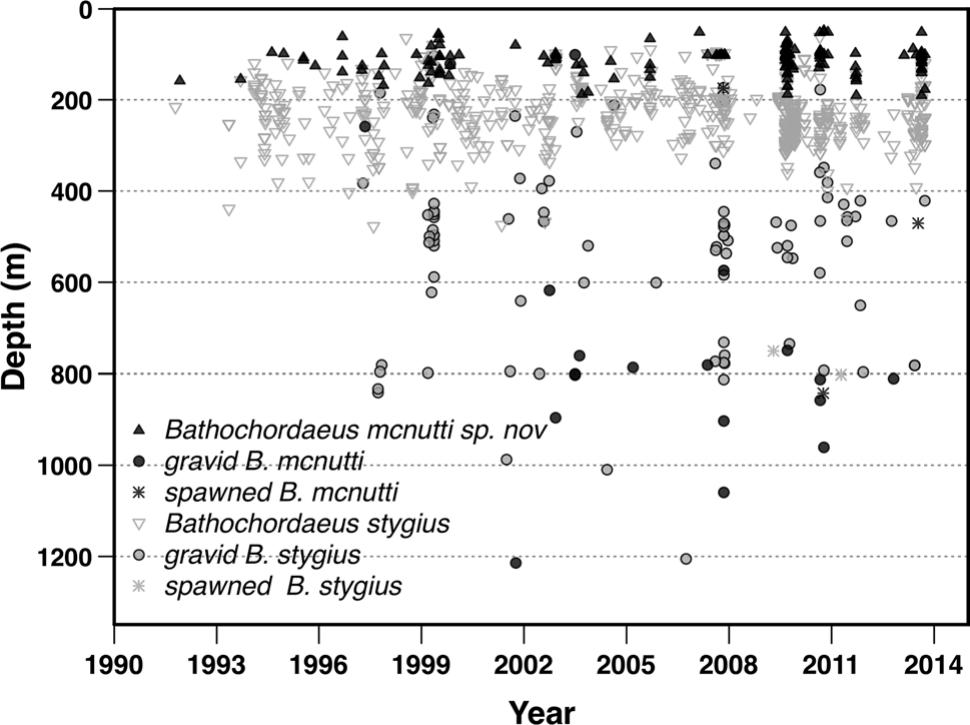
Depth distribution for *Bathochordaeus mcnutti* sp. nov. and *B. stygius*. Over MBARI’s 23-year video time series 64% of *B. mcnutti* (*n* = 161) have been found between 90 and 120 m. Although the depth distribution of the two species does overlap, only 28% of *B. stygius* (*n* = 672) were found above 190 m and 68% were observed between 200 and 300 m over the same time frame. For both species, the deepest animals found were always gravid or spent (had spawned their gametes) and were never found in houses (i.e., feeding)

From video, we have identified 833 non-gravid individuals to species level: 161 *B. mcnutti* and 672 *B. stygius*. From 1991 to 2014, the maximum depth observed for a non-gravid *B. mcnutti* was 189 m, with 64% of individuals found between 90–120 m (Fig. 8). In contrast, 28% of non-gravid *B. stygius* individuals were found above 190 m, while 68% of the individuals were found between 200–330 m.

Deeper occurring animals were more rare. Only 47 individuals from both species have been found in excess of 600 m. Of those, 42 animals were gravid and none were associated with a house (Fig. 8). The five non-gravid animals (three *B. mcnutti*, and two *B. stygius*) observed at depth were moribund, sinking, or swimming weakly downward. All were relatively large. None were associated with houses, and those that were observed closely appeared to have ruptured trunks. These animals appeared likely to have spawned in shallower waters and were either dead or dying as they sank back through the mesopelagic. In the laboratory, tails of spawned animals continued to beat reflexively, but weakly, several hours after the trunk had ruptured.

We observed a number of gravid and non-gravid *B. stygius* off the Oregon coast in summer of 2009. We did not encounter any *B. mcnutti* off Oregon or Washington; however, a single specimen was found at 86 m in the San Diego Trough off southern California (Latitude: 32.904325, Longitude: −117.782307). Animals from southern California, Oregon, and Washington are excluded from the depth comparison of *B. mcnutti* to *B. stygius* (Fig. 8).

### Molecular diagnosis

**Fig. 9:**
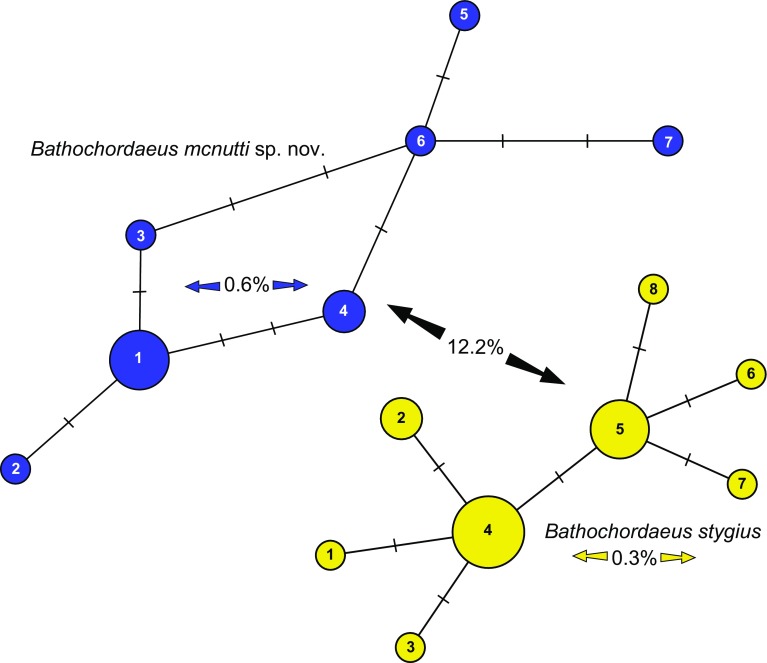
Molecular data support morphological observations of the differences between *Bathochordaeus mcnutti* sp. nov. and *B. stygius*. For the 400-base-pair region of the COI gene, the sequence difference between *B. mcnutti* (*n* = 11) and *B. stygius* (*n* = 17) averaged 12.2%. In comparison, the *B. mcnutti* haplotypes differed by an average of 0.6% within the 11 individuals, and *B. stygius* by only 0.3%

The 99.9% similarity of the 18S sequences (*n* = 3, one individual had one base-pair difference) confirmed what we had suspected based on morphological examinations: that blue-tailed giant larvaceans belong within the genus, *Bathochordaeus*. However, the 400-bp partial COI gene obtained from 17 individuals of *B. stygius*, and 11 individuals of *B. mcnutti* was more distinct. The genetic differences for COI between *B. mcnutti* and *B. stygius* are significantly greater than the haplotype differences found within each species (Fig. 9). For COI, the sequence difference between species averaged 12.2%. In comparison, the *B. mcnutti* haplotypes differed by an average of 0.6% within the group of 11 individuals, and the *B. stygius* haplotypes differed by an average of 0.3% for 17 individuals (Fig. 9).

## Discussion

Larvaceans are among the most common zooplankton, and their importance to the epipelagic, mesopelagic and benthic environments of the world ocean has been described in some detail (Alldredge 1981; Deibel 1988; Hamner and Robison 1992; Steinberg et al. 1994; Gorsky and Fenaux 1998; Hopcroft and Robison 1999; Robison 2004; Robinson et al. 2010). Larvaceans can also be among the most abundant mesozooplankton found as prey in the guts of many animals from medusae to fish (Gorsky and Fenaux 1998; Purcell et al. 2005). As they feed, small larvaceans like *Oikopleura dioica* filter relatively large quantities of water, concentrating food particles in the submicron range and making those particles more readily available to larger animals in the form of fecal pellets (Deibel 1998; Wilson et al. 2013). Alldredge (1977) showed that larger oikopleurids filtered larger prey and that relatively large oikopleurids like *Stegosoma magnum* can filter 20 L of water or more per day, enough water to have significant grazing impact. With trunk lengths of 2–3 cm, the largest *B. mcnutti* we have collected (Fig. 3) are close to an order of magnitude larger than the *S. magnum* studied by Alldredge (1977). Calculations made by Morris and Deibel (1993) showed that filtration rates of oikopleurids are a function of tail length, width, and beat frequency, from which Silver et al. (1998) calculated that *Bathochordaeus stygius* could filter approximately 250 l day^−1^. The mouth of an adult *Bathochordaeus* varies in size from approximately 1–2 mm in diameter and limits the size of the largest particles on which these animals can feed. Whether *Bathochordaeus* spp. are also able to feed upon submicron-sized particles is not known.

It is important to remember that some of the particles in the water filtered by oikopleurids remain on their filters, or “houses” (Gorsky et al. 1984). These particles include diatoms, ciliates, protozoans, fecal pellets, bacteria, as well as small invertebrates like copepods (Steinberg et al. 1994; Gorsky and Fenaux 1998; Silver et al. 1998). When abandoned, the discarded houses provide a ready way to rapidly transport carbon to the seafloor. However, because of their diaphanous nature, they are not well sampled by traditional methods such as sediment traps (Steinberg et al. 1994; Silver et al. 1998; Robison et al. 2005). We have observed hundreds of discarded houses, or “sinkers,” from *Bathochordaeus* spp. in the water column as well as on the seafloor, at times being consumed by a diverse group of animals such as ctenophores, munnopsid isopods, holothurians, and even the archaic cephalopod, *Vampyroteuthis infernalis* (Hoving and Robison 2012).

**Fig. 10:**
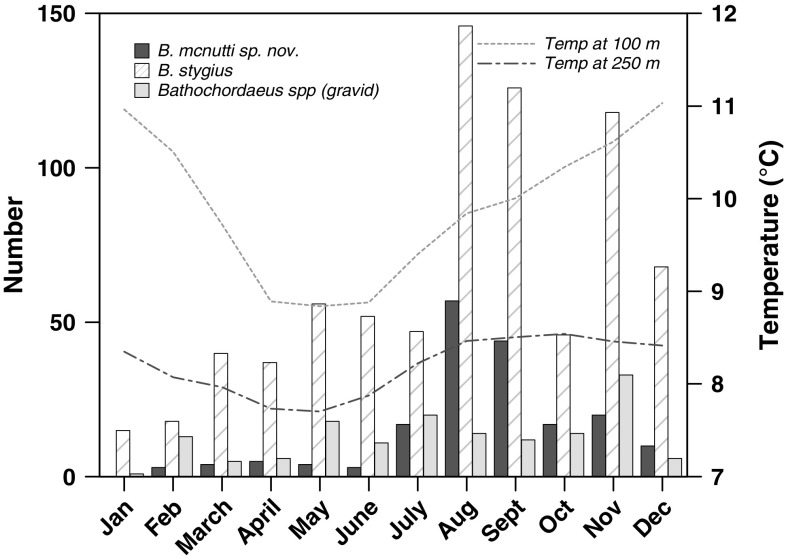
A twenty-three-year time series of collections and observations compressed into one calendar year. *Bathochordaeus mcnutti* sp. nov. is usually present in Monterey Bay for approximately 6 months. Typically, they begin to arrive in the summer as spring upwelling weakens and disappear sometime before the winter storm (Davidson) season begins. The abundance of both species peaks late summer to fall (September–November), but *B. stygius* tends to occur in higher numbers than *B. mcnutti* the rest of the year. Temperature data (averaged over the 23-year time series) show the cessation of upwelling during the summer, coincident with the highest abundance of *B. mcnutti*. The highest number of gravid individuals (both species) has been observed in late fall

Hydrography likely controls the presence/absence of *B. mcnutti* in Monterey Bay, where they occur only during part of the year. They begin to arrive in the summer and disappear in winter (Fig. 10). This timing corresponds to the Oceanic season, which occurs after spring upwelling weakens and the California Current moves onshore, often into Monterey Bay, but before the winter storm (Davidson) season begins (Pennington and Chavez 2000; Collins et al. 2003). As upwelling declines, warmer offshore surface waters ebb back toward the coast, perhaps bringing *B. mcnutti* with them. Blue-tailed larvaceans subsequently seem to disappear from Monterey Bay in the winter months and that timing coincides approximately with the onset of the northward-flowing Davidson Current sometime in November or December (Pennington and Chavez 2000). *Bathochordaeus mcnutti* may be a species that is found in warmer waters. We have not observed any during surveys made off the coast of Oregon but have recorded one in infrequent surveys made off southern California waters. More extensive sampling will need to be done to determine the geographical range of *B. mcnutti*.

*Bathochordaeus stygius* is also present in Monterey Bay during the fall and other mesopelagic fauna, micro- and macrozooplankton, have been linked to this biologically productive time (Robison et al. 1998; Kudela et al. 2008; Checkley and Barth 2009). However, *B. stygius* occurs in Monterey Bay throughout most of the year, perhaps because they live predominantly below (Fig. 9) the extent of the California Current, which shoals as it approaches shore (Castro et al. 2001).

Hydrography may also play a role in the life cycle of giant larvaceans and their seemingly unique (as a genus) habit of descending hundreds of meters while gravid (Fig. 8). Very little is known in general about the life histories of larvaceans, but their populations are generally thought to be tuned to event-scale (days to weeks), rather than seasonal-scale changes in environment (Deibel and Lowen 2012). *Bathochordaeus* spp., with their large size and growth to adulthood in water averaging <10 °C, may be an exception. Larvaceans are not known to have resting eggs or stages that over-winter, means by which some invertebrate taxa are able to survive lean times when their food is scarce (Strathmann 1987; Gorsky and Fenaux 1998). Over 23 years, more individuals of both species have been observed in the fall in Monterey Bay. These animals are large relative to specimens collected earlier in the year, and often they are gravid (Fig. 10). Presumably, they have been feeding and growing throughout the upwelling season and into the Oceanic season. Descending to colder waters where gametes will develop more slowly, before ascending to finally spawn, may be a reproductive strategy similar to over-wintering in that it could allow *Bathochordaeus* spp. to avoid some of the pitfalls of the Davidson season, with its northward, onshore, downwelling current, and relatively nutrient-poor waters (Pennington and Chavez 2000; Collins et al. 2003).

Molecular data support the morphological findings in this study. Mitochondrial DNA from larvaceans (and urochordates in general) is difficult to sequence using traditional DNA barcoding primers for the cytochrome *c* oxidase subunit I region (Hirose and Hirose 2009; Bucklin et al. 2011); however, an increasing number of tunicate COI sequences using the degenerate primers of Hirose et al. (2009) are published in GenBank for salps and tunicates.

DNA barcoding is an important tool in the determination of biodiversity in oceanographic studies (Bucklin et al. 2010). However, largely because of the high rates of mutation and genetic simplification, tunicates in general have proven difficult to study using molecular tools (Denoeud et al. 2010). As of May 2015, there are only two larvacean species with COI sequences in GenBank, both oikopleurids: *Oikopleura dioica* and *Oikopleura intermedia*. In order to make comparisons between species of larvaceans, more sequences need to be published and made available. Because these animals are fragile and difficult to collect intact by conventional methods, access to more molecular sequences will be instrumental in identifying new species of larvaceans. Molecular data in conjunction with morphological traits, ecological data, and in situ observations will provide more opportunities for species discoveries in these abundant and ecologically important animals.

## Electronic supplementary material

Below is the link to the electronic supplementary material.

Supplementary material 1 (M4 V 70766 kb)
